# Crosstalk between p53 and TGF-**β** Signalling

**DOI:** 10.1155/2012/294097

**Published:** 2012-03-28

**Authors:** Rebecca Elston, Gareth J. Inman

**Affiliations:** Division of Cancer Research, Medical Research Institute, Ninewells Hospital and Medical School, University of Dundee, Dundee DD1 9SY, UK

## Abstract

Wild-type p53 and TGF-**β** are key tumour suppressors which regulate an array of cellular responses. TGF-**β** signals in part via the Smad signal transduction pathway. Wild-type p53 and Smads physically interact and coordinately induce transcription of a number of key tumour suppressive genes. Conversely mutant p53 generally subverts tumour suppressive TGF-**β** responses, diminishing transcriptional activation of key TGF-**β** target genes. Mutant p53 can also interact with Smads and this enables complex formation with the p53 family member p63 and blocks p63-mediated activation of metastasis suppressing genes to promote tumour progression. p53 and Smad function may also overlap during miRNA biogenesis as they can interact with the same components of the Drosha miRNA processing complex to promote maturation of specific subsets of miRNAs. This paper investigates the crosstalk between p53 and TGF-**β** signalling and the potential roles this plays in cancer biology.

## 1. Introduction

Transforming growth factor beta (TGF-*β*) is a pleiotropic cytokine responsible for the regulation of nearly every human cell type. Under normal conditions, TGF-*β* functions in a context specific manner to maintain tissue homeostasis largely via transcriptional regulation of genes involved in proliferation, cell survival and cytostasis, differentiation, cell motility and the cellular microenvironment [1–8]. Unsurprisingly, the function of this habitual tumour suppressor is commonly found to be perturbed in cancer cells via receptor and pathway component mutations and oncogene crosstalk [1, 4, 9]. Paradoxically, TGF-*β* can also act as a tumour promoter as tumourigenesis progresses [1, 3, 4, 10–12]. 

**Figure 1:**
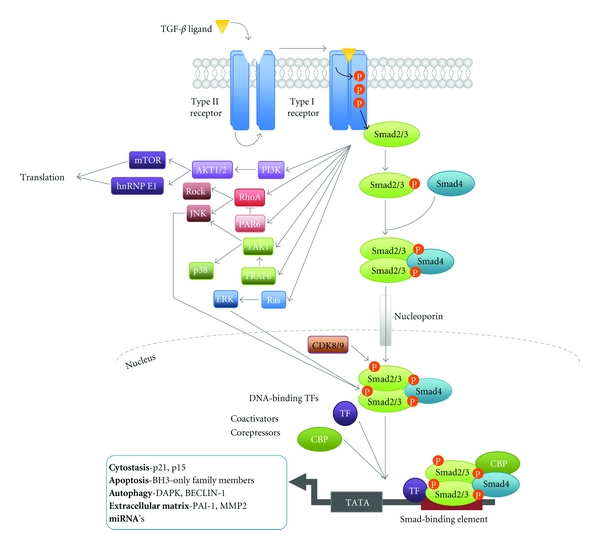
Canonical and Noncanonical TGF-*β* signalling. Initiation of the TGF-*β* signalling cascade occurs via binding of active TGF-*β* ligand to the TGF-*β* type 2 receptor (TGF-*β*RII) [1, 5]. Once bound TGF-*β*RII is then able to activate its partner the TGF-*β* type 1 (TGF-*β*RI)/ALK5 receptor via phosphorylation [1, 14]. Phosphorylation of TGF-*β*RI results in a conformational change by which the kinase repressive N-terminal GS domain is flipped to act as a docking site for Receptor Smad (R-smad) proteins for example, Smad2 and 3 and in turn facilitates signal transduction by activation of the catalytic kinase domain [1, 16]. TGF-*β*RI phosphorylates Smad2 and 3, which associate with their co-smad Smad4 to form the active Smad complex, which accumulates in the nucleus via nucleoporin-mediated transport [5, 40]. Phosphorylation acts to inhibit the constant nucleocytoplasmic recycling of Smads resulting in nuclear accumulation [41]. Smads associate with DNA via binding at target gene DNA-Smad Binding Element's (DNA-SBE), with a optimal conserved sequence of 5′-CAGAC-3′ [17, 42]. However, the Smad complex has only relatively weak DNA-binding affinity. Thus, association with numerous DNA-binding transcription factors for example, Zinc-fingers, homeobox and bHLH families, coactivators (e.g., CBP-300), corepressors (e.g., RBL1) and chromatin remodeling factors (e.g., Histone Deacetylase (HDAC)) allows the complex to achieve specific cell responses [17, 42, 43]. In addition activated TGF-*β*RI can also activate multiple noncanonical pathways. These Smad independent pathways can function autonomously to achieve a wide array of cellular responses in a transcription-independent manner [21]. In addition activation of the JNK, ERK, and CDK8/9 pathways regulate Smad linker phosphorylation to regulate Smad activity [22, 44].

TGF-*β* signalling is mediated through binding of a TGF-*β* isoform (TGF-*β*1, 2 or 3) to a transmembrane heterotetrameric complex of the serine/threonine kinase receptors T*β*RII and T*β*RI [13–15]. Activation of the TGF-*β* receptors results in the downstream phosphorylation of transcription factors Smad2 and 3, which subsequently dissociate from the receptor before binding their constitutive co-smad, Smad4 (Figure 1) [1, 5, 16–18]. The active Smad complex accumulates in the nucleus where it functions to regulate transcription of a myriad of target genes including p21 and proapoptotic BH3-only members of the BCL2 family (Figure 1) [6, 8, 19, 20].

As well as the activation of the Smad dependent signalling pathway, TGF-*β* can also regulate non-Smad pathways such as the JNK/p38, Ras-ERK, PI3K/Akt, and RhoA pathways [2, 21, 22]. These pathways can then act to regulate many cellular proteins including Smad interacting coactivators and corepressors and thereby contribute to the context specificity of TGF-*β* signalling (Figure 1) [2]. Regulation of biological processes by TGF-*β* signalling is consequently brought to fruition by integration of these signalling cascades with other physiological pathways operating in the target cell.

Renowned as the “Guardian of the Genome” p53 has a critical role in maintaining the genetic integrity of proliferating cells thereby preventing malignant transformation [23–25]. p53 acts primarily as a transcription factor [26]. In response to stress signals for example, genotoxic stress, DNA damage, oncogene activation, and hypoxia, p53 is stabilised resulting in its accumulation and subsequent recruitment to p53 binding sites in chromatin [23, 27–29]. Once chromatin bound, p53 promotes transcriptional activation of numerous target genes responsible for apoptosis for example, BH3-only family members and cytostasis for example, p21, [30–33]. More recently p53 has been found to have a much broader range of functions stimulating DNA repair, cell adhesion, cell motility, membrane functioning, and metabolism [23, 34].

With its central role in tumour suppression, p53 is the most commonly mutated gene in cancer with over 50% of tumours expressing a mutant variant [35, 36]. Mutations are particularly prominent in the central DNA binding domain with 74% occurring here, 30% of which occur at six hotspot codons [35]. Mutation of p53 can result in a gain of function acting to promote tumourigenesis [23, 36, 37]. Additionally once mutated p53 may have a dominant negative effect over its wild-type counterpart acting to induce chromosomal instability, a feature of tumour progression, and suppress genes involved in cell cycle control, apoptosis, and DNA repair pathways [36, 38, 39].

## 2. TGF-***β*** and Wild-Type p53 Pathway Convergence

 Under normal phenotypic conditions, both TGF-*β* and activated p53 act as gene-specific transcription factors to each regulate a multitude of gene targets producing tumour-suppressive effects. Due to the broad ranging nature of these proteins, overlap of cellular functions occurs in the regulation of cytostasis, apoptosis, and autophagy indicating several potential points of convergence. The initial link between the wild-type p53 and TGF-*β* pathway was proposed in 1991 by Wyllie et al. Inactivation of wild-type p53 by the SV40 virus resulted in loss of response to TGF-*β* treatment implying that loss of p53 may prelude resistance to the tumour-suppressive effects of TGF-*β* [55]. More recently several studies have demonstrated the convergence of p53 and Smad signalling pathways.

### 2.1. Unearthing the Interaction

**Figure 2:**
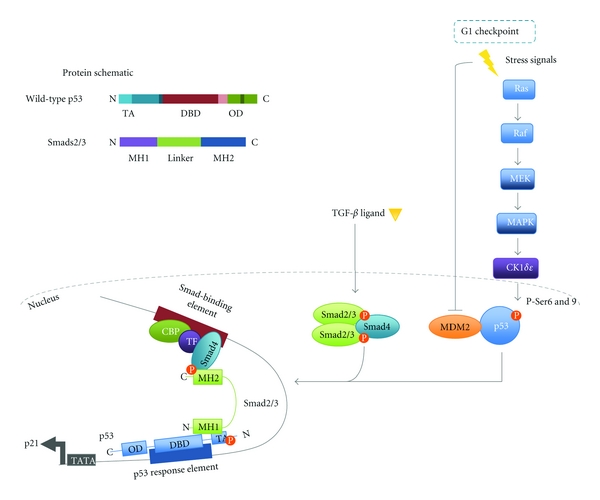
Coregulation of gene transcription by p53 and Smad complexes. In the absence of cellular stress p53 is maintained at a low concentration by its negative regulator MDM2 [27, 28, 45]. MDM2 acts to poly-ubiquitinate p53, which targets the tumour suppressor to the proteasome for degradation. In response to DNA damage p53 is phosphorylated at defined Ser/Thr residues resulting in its stabilisation and dissociation from MDM2 [29]. In addition signalling via the Ras/MAPK pathway CK1*δ*/*ε* can also result in the activating phosphorylation of p53 [46]. Activated wild-type p53 can act synergistically with Smads to increase the transcription of a subset of genes for example, p21, PAI-1 [46, 47]. The model depicted was originally proposed by Piccolo and colleagues and elegantly demonstrates how p53 and Smads may interact [46–48]. For synergism to occur, the target gene must possess both a DNA-SBE and a p53 response element (RE) to which the activated Smad complex and p53 bind respectively. Once bound at their respective sequences a direct interaction between Smads and p53 may occur in which the N-terminal MH1 domain of Smads2/3 binds the N-terminal transactivational domain (TA) of p53 [46–48]. Association within the gene promoter acts to maximally induce gene transcription.

p53 has been identified as a gene-specific partner for Smads important for the formation and stabilisation of Smad-DNA complexes. The suggestion of such a partnership was first revealed by functional assays in Xenopus embryos searching for modulators of TGF-*β* family signalling regulated processes during early development [47, 56]. In both screens, p53 was found to regulate development and that this activity required Smad proteins operating through regulation of specific target genes such as Mix2 [47, 56]. Upon TGF-*β*/Activin, signalling FAST-1 forms a complex with Smads at the Mix2 promoter thereby facilitating gene transcription [57]. Using the Xenopus embryo model system, Cordenonsi et al. demonstrated that in the presence of a p53 isoform (p53AS) along with FAST-1, TGF-*β* induces a robust increase in the transcription of Mix2 and importantly this effect was diminished upon p53 knock down [47]. In the same study, Cordenonsi et al. used the mammalian HEK293T cell line to further elucidate the mechanism of this p53-Smad interaction. Both Smad2 and 3, but not Smad4, were found to directly bind immobilised wild-type p53 and p73 [47]. Specifically, binding occurs through the phosphorylated N-terminal domain of activated p53 and the N-terminal MH1 domain of Smad2/3 with the C-terminal MH2 domain free to interact with Smad4 [47]. Coupling of these proteins is dependent upon the phosphorylation of p53 at Serine 6 and 9 of the N-terminal transactivational domain. Phosphorylation of these residues typically occurs in response to DNA damage. However, the Ras/MAPK cascade acting via casein kinase 1 (CK1) *δ* and *ε* can also promote this phosphorylation as demonstrated in mammalian H1299 cells [46]. Crucially, inhibition of the Ras/MAPK effector MEK abrogated p53 phosphorylation and subsequently diminished induction of key TGF-*β* cytostatic genes p21 and p15 thus indicating that p53 serves to integrate crosstalk between Ras/MAPK and TGF-*β* signalling [46]. This interaction of p53 with Smads 2 and 3 occurs in a TGF-*β* dependent fashion [47]. However, to induce a robust increase in TGF-*β* mediated gene transcription p53 must contact its own cognate site within the gene promoter [46–48]. Deletion or point mutation of the DNA binding domain of p53 blocks its biological activity as demonstrated in Xenopus assays and thereby impedes complex formation of the protein with Smads [47, 48, 56]. Thus Smad2/3 may act as a bridge between p53, bound at the p53 binding-element and the Smad complex, bound at the TGF-*β* responsive-element, allowing synergistic activation of transcription (Figure 2) [46–48].

Synergism between p53 and TGF-*β* occurs only with a subset of TGF-*β* target genes with p53 presence having no effect on the inducibility of others for example, TIEG-1/2 [47]. In spite of this, bioinformatic screening of 800 TGF-*β* target regulatory sequences versus a genomic database of putative p53 DNA-binding sites revealed in excess of 200 genes could be potentially coregulated [48]. However, clustering analysis of these genes predicted only growth inhibitory and extracellular matrix functions to be under joint regulatory control [48]. In support of this, TGF-*β* target genes such as p21 and p15 (growth inhibition), PAI-1 and MMP2 (extracellular matrix) require wild-type p53 for full activation [46, 47]. Interestingly, in the context of cytostasis wild-type p63 can compensate for p53 mutation having a functional overlap in regulating at least the p21 gene which is required for cell cycle arrest [47]. A prime example is the TGF-*β* responsive HaCaT cell line, which is p53 mutant but expresses high levels of p63. Following p63 directed siRNA p21 induction by TGF-*β* was reduced [47]. However more recently, mutant p53 and TGF-*β* have been found to complex and induce TGF-*β*-induced metastasis suggesting coregulation of additional cell responses may also occur [53] (see below).

### 2.2. Smad and Wild-Type p53 miRNA Crossover

MicroRNAs (miRNAs) are small, noncoding RNAs that regulate protein expression by inhibiting translation and increasing degradation of mRNA [58–60]. Primary (pri-) miRNA is transcribed as a long transcript, which folds back to form a hairpin structure [61]. Inside the nucleus DROSHA processes pri-miRNAs cleaving the 5′ Cap and 3′ Poly (A) tail to form precursor (pre-) miRNAs which are then transported to the cytoplasm via exportin 5 where cleavage by DICER produces mature miRNAs [58, 59, 62]. Once matured miRNAs are incorporated into the RNA-Induced Silencing Complex (RISC), which positions the miRNA with its target mRNA at the 3′UTR to mediate inhibition [58–60, 63].

The downregulation of mature miRNAs either at the genomic level, by epigenetic modifications or by impaired biogenesis is observed in human malignancies resulting in the promotion of cellular transformation and tumourigenesis [64–66]. Extracellular signals can influence the maturation of specific miRNAs, for example TGF-*β* and p53 have been found to positively regulate their biogenesis [49, 50]. Such positive regulation on biogenesis may act in a tumour suppressive manner for example, TGF-*β* and p53 induce miR-215, which acts to induce growth arrest and decrease cell proliferation [51, 67, 68]. Conversely, a positive regulation on biogenesis may also lead to tumour progression for example, TGF-*β* increases miR-21 maturation [51], which can act to inhibit PTEN and Sprouty 1 key negative regulators of the Akt and Ras/MAPK pathways allowing their aberrant activity [69, 70].

Both p53 and TGF-*β* can act transcriptionally to regulate the expression of miRNAs by binding at their response elements within target promoters. It is plausible that p53 and TGF-*β* may converge via the p53-Smad interaction as earlier described to synergistically regulate miRNA transcription. However, whether such an interaction at miRNA promoters does in fact occur is yet to be described.

**Figure 3:**
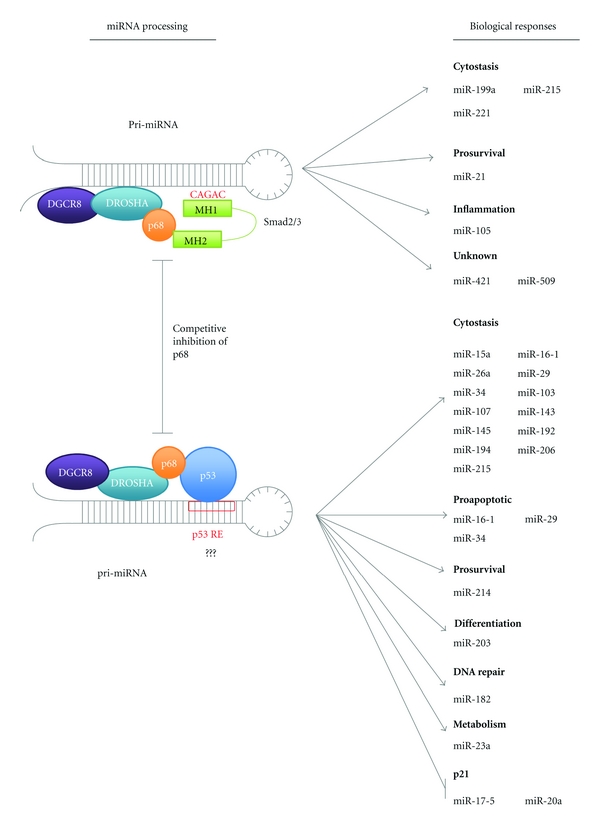
Potential overlapping functions of TGF-*β* and p53 in microRNA processing. Smads and p53 act to increase the posttranscriptional maturation of a subset of miRNAs via direct binding of the DROSHA-associated helicase p68 [49–52]. These miRNAs have crucial roles in tumour suppression acting in cytostasis and DNA repair. Interestingly pro-survival miRNAs are also upregulated indicating a possible mechanism for protumourigenesis by inhibition of key tumour suppressors. However, upregulation of these prosurvival miRNAs may also facilitate the induction of senescence, protecting cells from cell death induced by tumour suppressor genes. In addition to acting as a molecular tag to direct DROSHA activity binding of Smads and p53 may act to promote p68 helicase activity, which could act to induce conformational changes in pri-miRNA structure making it accessible for DROSHA cleavage. As proposed by Davis et al. competition may occur between Smads and p53 for binding to p68 upon which binding of one protein results in the inhibition of p68 association with other [51]. Binding of Smads to microRNAs is mediated by association with CAGAC Smad DNA-binding-like elements (SBEs) and a similar p53 response-element- (RE) mediated mechanism may also occur for p53. Potential RE and SBE sequence overlap may also occur adding a potential further layer of cross-regulation.

In response to TGF-*β* signalling miRNA microarray profiling of PASMCs revealed mature levels of 20 miRNAs, including miR-21, that were induced in excess of 1.6-fold [51]. Of these miRNAs analysis by Davis et al. revealed 85% contained a conserved stem sequence (CAGAC) homologous to that of the SMAD-binding element present in DNA (Figure 3) [51]. This RNA Smad-binding element (R-SBE) was absent in non-TGF-*β*-induced miRNA's [51]. In C3H10T1/2 cells mutation of more than 2 bp in the R-SBE sequence abolished the production of these mature miRNAs revealing that Smads must associate with these miRNAs directly for upregulation to occur [51].

Posttranscriptionally Davis et al. defined how Smad's enhance miRNA maturation. Following TGF-*β* treatment of PASMC cells the induction of both pre- and mature miR-21 was observed, however, no change in expression of pri-miR-21 was seen indicating TGF-*β* components must act posttranscriptionally with DROSHA [49, 51]. Use of recombinant GST-Smad fusion proteins demonstrated that the MH1 domain of Smads is responsible for binding of the R-SBE in miRNAs with this domain binding and pulling down 18-fold more pri-miR-21 than GST, whereas the MH2 domain did not bind at all [51]. The association of Smads with miRNAs is unable to facilitate their maturation, instead Smad binding is proposed to act as a molecular tag-promoting interactions with the processing machinery [51]. Both Davis et al. and Warner et al. reported that the MH2 domain of TGF-*β* Smad2/3 (but not Smad4) interacts with the RNA helicase p68 (Figure 3) [49, 52]. p68 is part of the DROSHA multicomponent processing complex and may function to recognise and bind specific pri-miRNA structures and thus localise DROSHA for cleavage [71]. Further investigation in PASMCs by Davis et al. revealed that siRNA of p68 inhibited the TGF-*β*-mediated induction of pre- and mature miR-21 thereby demonstrating it's essential role in potentiating DROSHA processing [49]. The enhancement in miRNA maturation may facilitate TGF-*β* tumour-promoting functions. For example miR-21 is highly expressed in several breast tumour cell lines including, MCF7 and MDA-MB-468 cells where it acts to suppress the apoptotic tumour suppressor programmed cell death 4 (PDCD4) [49].

In response to DNA damage activated wild-type p53 can transcriptionally upregulate miRNAs via direct binding at target promoters for example, miR-34. In addition activated p53 can also act post-transcriptionally to promote the maturation of a subset of miRNAs important in the regulation of cell cycle and proliferative genes for example, K-Ras and CDK6 [50]. Notably these miRNAs are distinct from those induced by TGF-*β* having no R-SBE. Suzuki et al. determined that like the Smads p53 interacts with the RNA helicases p68 and p72 to facilitate DROSHA processing (Figure 3) [50]. Mutation of p53 also results in decreased miRNA maturation, however, as mutant p53 still associates with p68 suppression must occur in a transcription-dependent manner [50].

As both p53 and Smads can interact with the same components of the Drosha complex it is possible that activation of these transcription factors may result in competition for Drosha complex components and may therefore cross-regulate each other's responses. Indeed Davis et al. noted that maturation of Smad-regulated miRNAs is enhanced in cells with loss of p53 function, for example the mutant p53 cell line MDA-MB-468 versus the wild-type p53 PASMCs [51]. 

## 3. Inhibition of TGF-***β*** Tumour Suppressor Function by p53 Mutation

### 3.1. Transcriptional Activation of TGF-*β* Target Genes

p53 mutants have been found to affect numerous stages of the TGF-*β* pathway; by repressing of the TGF-*β*RII gene, delaying or reducing phosphorylation of Smad2 by TGF-*β*RI, and interfering with Smad2/3 and Smad4 association and inhibiting Smad translocation to the nucleus [72]. By affecting the TGF-*β* pathway Smad-dependent transcription of target genes is ablated and thus TGF-*β* mediated cellular responses. In addition p53 mutation can result in a loss of DNA-binding capacity thereby inhibiting coupling with Smad's at gene-specific promoters for example, Mix2, PAI-1, and p21 and thus transcriptional induction of these genes is diminished [46, 72]. p21, a cyclin-dependent kinase inhibitor, acts to induce a functional G_1_ arrest via the inhibition of downstream cell cycle regulators CDK4/Cyclin D1 and CDK2/Cyclin E. Inhibition results in the hypophosphorylation of the retinoblastoma (Rb) protein preventing its release from E2F, a key transcription factor for DNA replication and thus blocking G_1_ to S phase progression. Plasminogen activator inhibitor 1 (PAI-1) acts to inhibit both the degradation of collagen and the catalytic activation of matrix metalloproteinases (MMP) 1 and 10 [73]. When active, these MMPs play a crucial role in the invasion of malignant cells across the basal lamina. The degradation of collagen also mediates this invasion as well as invasion into local blood vessels allowing cells to metastasise. Consequently diminished activation of these genes may aid tumourigenesis inducing limitless replicative potential from a failure to growth arrest by p21 and through MMP activity tissue invasion and metastasis both key hallmarks of cancer [74].

Inactivation of p53 results in aberrant behaviour of cancer cells and may therefore represent a mechanism by which loss of TGF-*β* tumour suppression occurs [75]. In support Cordenonsi et al. found that reintroduction of the wild-type p53 isoform p53AS in conjunction with Smad2 induced a TGF-*β*-mediated cytostatic response within the normally unresponsive p53 null SAOS-2 cell line [47].

### 3.2. Metastasis

**Figure 4:**
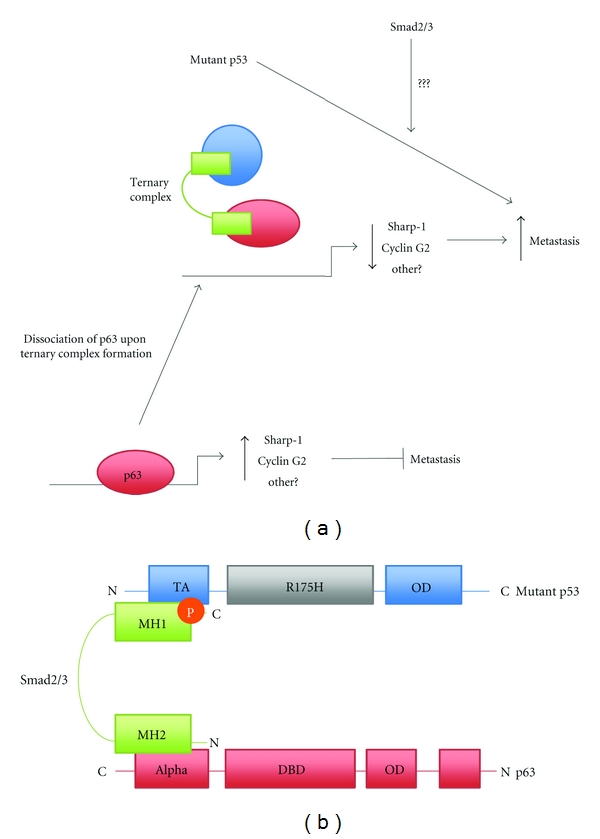
Regulation of metastasis by mutant p53 and Smads. (a) p63 has a crucial role in the transcriptional activation of genes which function in the inhibition of metastasis. Mutant p53 can act to inhibit the anti-metastatic functioning of p63 but is dependent upon the formation of a ternary complex with Smads2/3 as described below [53]. Formation of a ternary complex inhibits the binding of p63 to the DNA at target gene promoters thereby inhibiting transcription of these key antimetastatic genes and subsequently increased metastasis of malignant cells is observed [53]. In addition mutant p53 can promote invasion and metastasis independently. For example mutant p53 can suppress p63 in the context of integrin recycling [54]. It is yet unknown whether Smads are involved for these other routes of induction. (b) Mutant p53 is able to inhibit p63 activity by the formation of a ternary complex. Formation of this complex is dependent on receptor Smad2/3 acting as a molecular bridge between the proteins. The C-terminal MH1 domain of Smads2/3 binds the transactivational (TA) domain at the N-terminus of mutant p53 [53]. The N-terminal MH2 domain binds at the C-terminal alpha domain of p63 [53]. Association of p63 in this complex inhibits its capacity to bind DNA thus blocking transcriptional functions of the protein.

Recently Adorno et al. demonstrated that the mutational status of p53 determines the nature of the cellular response to TGF-*β*. Introduction of wild-type p53 into p53 null H1299 cells resulted in a TGF-*β*-induced growth arrest via p21 [53]. In contrast reconstitution with mutant p53 caused cells to change from an epithelial to mesenchymal morphology, enabling a promigratory TGF-*β* response [53]. Under normal conditions metastasis is inhibited by the p53 family member p63 which acts to transcriptionally upregulate key metastatic suppressor genes for example, Sharp-1 and Cyclin G2 [53]. In conjunction with Smads mutant p53 can usurp p63 functioning via ternary complex formation. For example in metastatic D3S2 carcinoma cells, TGF-*β* treatment resulted in complex formation of nearly all the p63 with mutant p53 thereby facilitating a TGF-*β* metastatic response. Formation of this complex was found to be dependent on Smads becoming undetectable post-transfection of Smad2/3 siRNA in HaCaT cells. To determine the structural association of Smads in this complex GST-pull down experiments were carried out using immobilised GST-Smad3 and structural isoforms of p63 resulting in the identification of two Smad interaction sites within p63 at the transactivational and alpha domains [38]. Importantly the vast majority of p63 is expressed as the ΔNp63 isoform, which lacks an N-terminal transactivation domain [38]. As a result Adorno et al. hypothesised a structure in which R-Smads acting as a bridge between the C-terminal alpha domain of p63 and the N-terminal domain of p53 via respective binding of Smad MH2 and MH1 domains (Figure 4) [53].

Similarly increased invasion is observed in the context of integrin recycling in which mutant p53 expression again inhibits p63 functioning. Here inhibition of p63 promotes the association of Rab-coupling protein with *α*5*β*1 integrin which acts to chaperone the integrin to the plasma membrane where it then acts to promote tumour cell motility [54]. There is no evidence that these processes are influenced by TGF-*β* signalling and Smad interactions but this warrants further investigation.

### 3.3. miRNA Biogenesis

In cells harbouring mutant p53 TGF-*β* stimulation results in greater induction of TGF-*β*-regulated miRNAs [51]. Davis et al. proposed that Smads and p53 might directly compete for p68 binding and hence loss of p53/p68 interaction may release more p68 for Smad-directed miRNA maturation (Figure 3). In addition to this proposed mechanism of p53/Smad crosstalk in miRNA biogenesis, it is also possible that competition between p53 and Smad complexes for direct RNA binding in the Pri-miRNA seed sequence may occur. Smads regulate a subset of miRNAs via interaction with the SBE consensus AGAC sequence. p53 regulates target genes via binding to the p53 RE which consists of 2 motifs, 5′-PuPuPuC(A/T)(T/A)GPyPyPy-3′, separated by a ≤13 bp spacer [76]. Computational analysis has previously revealed these binding sites to be conserved at the miR34b/c promoters [77]. By assuming a 5′ sequence of 5′-AGAC-3′, this motif has the potential to overlap with the core consensus at Smad-binding-elements [78]. In this case, binding of wild-type p53 at its cognate DNA may result in the displacement of Smads thereby inhibiting Smad-p68 interaction or vice versa. This intriguing possibility and the consequence of p53 mutation on this potential crosstalk warrants future investigation.

## 4. Concluding Remarks

Establishment of TGF-*β*-p53 pathway crossover brings new complexity to the regulatory functioning of these proteins. Currently TGF-*β* and p53 convergence has only been found in cytostasis, extracellular matrix functioning, and metastasis [48, 53]. However, it will be interesting to ascertain whether such crossover occurs in other TGF-*β* and p53 functions namely apoptosis, proliferation, and autophagy and if such responses are dependent on the presence or phenotypic status of both proteins.

The recent study of Adorno et al. demonstrated how the mutational status of p53 can interfere with TGF-*β* switching it's functioning from tumour suppressive to protumorigenic [53]. In concurrence Davis et al. shows mutant p53 confers the ability of TGF-*β* to promote miRNA maturation for example, miR-21 which can act to inhibit tumour suppressors such as PDCD4 [49]. Such findings suggest that mutant p53 may potentiate tumour-promoting functions of TGF-*β* thus allowing cancer cells to proliferate and invade leading to advanced tumours.

TGF-*β* has been implicated in the induction of EMT in breast cancer stem cells and plays an essential role in maintaining the pluripotency of these stem cells [79]. In the Adorno et al. study p63 acts to suppress TGF-*β* induced EMT [53]. However, in the presence of mutant p53 formation of the ternary complex with p63 in conjunction with Smads acts to inhibit this p63 suppression facilitating TGF-*β* induced EMT [53]. This raises the interesting question of whether TGF-*β*/Smads and p53 family members may also cooperate to regulate tumour stem cells.

Further complexities between crosstalk of p53 family members and their multiple splice variants [80] with TGF-*β* signalling pathways are likely to be unveiled in the near future. For example in MCF10A cells expression of ΔNp63*γ* results in TGF-*β*-dependent EMT and subsequently increases the expression of both TGF-*β* and downstream Smads2/3/4 [81]. A systematic evaluation of the effects of activation of the p53 family and TGF-*β* signal transduction pathways in different biological situations is likely to shed considerable light and add further intrigue into the crosstalk between these fundamentally important regulators of biology.
